# Fractures and Dislocations

**Published:** 1895-08-03

**Authors:** 


### FRACTURES AND DISLOCATIONS.

(*Continued from page* 224.)

Luke Freer[13] has recently divided, with good results, the tendons of the palmaris longus, flexor carpi radialis, flexor sublimis, and flexor profundus digitorum in a case of contraction of the fingers, with anæsthesia, after fracture of the bones of the forearm. A simple and easily constructed splint for the treatment of Colles' fracture is advocated by Doyle.[14] Two or three thicknesses of cloth or flannel are cut to the proper shape, dipped in plaster of Paris cream, and secured by a roller to the front of the normal forearm, with the hand slightly flexed. After the plaster has set, it is removed, and allowed to dry thoroughly. The fractured arm after reduction is placed in the splint, which readily retains the bones in their normal position by the aid of a roller bandage, smoothly but not tightly applied, no posterior splint being required. Hennequin relates[15] two cases which, in his opinion, fully justify the performance of osteotomy, hitherto rarely performed, in instances of deformity of the radius after fracture of its lower end, and associated with the functional disturbance of the hand.

**Lower Extremity.**—A case of recovery with probable bony union after fracture of the neck of the femur in the eighty-eighth year of age is reported by W Pugin Thornton.[16] The patient, however, was only able to walk (at the time of the report) with the aid of crutches. During the past two years three instances illustrating the serious after effects of impacted extra-capsular fracture of the neck of the femur having come under the notice of F. A. Southam,[17] this surgeon has—contrary to the teaching of the text-books—recently in one case broken down the impaction at once. Under anæsthesia, the eversion and shortening by this means having been overcome, the fracture was treated in the ordinary way by extension and a long straight outside splint. The fracture readily united with the limb in the corrected position, no deformity remaining. Gibney[18] advocates arthrotomy in traumatic (as well as congenital) dislocations of the hip in children. The results, however, of the two cases operated upon by him by Hoffa's method do not appear to have been very brilliant. An interesting specimen of oblique fracture traversing the lower end of the femur in an oblique direction downwards and forwards and terminating just above the articular surface of the condyles is figured by Howard Marsh.[19] It was taken from a lady, aged 52, on whom amputation was performed eight weeks after the injury, for irreducibility of the fragments due to transfixion by the upper fragment of the broad tendinous expansion of the quadriceps just above the patella. A prolonged dissection and replacement of the fragments, which would have been undertaken in a young subject, involved too serious a risk in this case. Marsh gives illustrations of similar fractures and displacements from the museum of St. Bartholomew's Hospital, but in one specimen the upper fragment is displaced backwards into the popliteal space instead of forwards. In young children it is possible, according to a specimen recently dissected by E. E. Ware,[20] for the leg to be torn off through the ligamentous structures of the knee joint, rather than for the femur to give way at its lower end. The case alluded to was in a boy aged seven years, whose leg became entangled in the spokes of a four-wheeled cab. There was little hæmorrhage from the stump, the femoral artery being occluded by torsion. Amputation of the thigh was successfully performed. From a paper by Meisenbach[21] on resection of the knee joint for separation of the lower epiphysis of the femur from disease or injury, it appears that the lesion occurs as the result of injury in about $\frac{1}{5}$ to $\frac{1}{4}$ of all reported cases. Tyson[22] publishes a fatal case of pyæmia, resulting from compound comminuted fracture of the tibia, the skin being only slightly broken. It occurred at a date when the immense importance of antiseptic or aseptic surgery was not fully recognised. Tyson thinks that the time has come when all compound fractures should be treated by uniting the ends of the bone, the wound being thoroughly irrigated. Five cases of apparently hopeless fractures of the ankle joint are reported by Balch[23] as terminating in very good results. A posterior wire splint was employed, which allowed easy adjustment of the fragments from time to time as the swelling went down, while the foot was firmly held in the right position. Dry sterilized gauze is to be preferred, but in certain cases a wet antiseptic dressing will hasten the separation of sloughs. Criekx and Van Engelen's[24] experiments and clinical observations upon the regeneration of osseous tissue show that the transplanted portion of decalcified bone not only hastens, but also regulates osteogenesis. It serves as a support to the periosteal muff, preserves its form, and permits of the formation within it of a bone similar in shape and proportions to the original bone. The portion of bone transplanted and the wound should be absolutely aseptic.

[13] Lancet, Dec. 1, 1894. [14] Therapeutic Gazette, Oct. 15, 1894. [15] Revue de Chirurgie, Sept., 1894. [16] Lancet, Jan. 12, 1895. [17] Lancet, Nov. 17, 1894. [18] Annals of Surgery, Dec., 1894. [19] The Practitioner, Jan., 1895. [20] Lancet, Jan. 12, 1895. [21] N. Y. Med. Record, Dec. 15, 1894. [22] Clinical Journal, Jan. 9, 1895. [23] Boston Med. and Surg. J., Aug. 30, 1894, and Ther. Gazette, Oct. 15, 1894. [24] Journal de Med. de Chir. et de Pharm., ann. tome, iii. fasc. 3 B. 1894., and Am. J. of Med. Sci., Jan. 1895.

